# Relationship between personality traits and health behavior among working women in Japan

**DOI:** 10.1016/j.pmedr.2021.101691

**Published:** 2021-12-27

**Authors:** Miho Satoh, Naoko Sato, Akiko Fujimura

**Affiliations:** a Department of Fundamental Nursing, Yokohama City University, Yokohama, Kanagawa, Japan; b Department of Clinical Nursing, Fukushima Medical University, Fukushima, Japan; c Department of Nursing, Tokyo Healthcare University, Tachikawa, Tokyo, Japan

**Keywords:** CI, confidence interval, MHLW, Ministry of Health, Labour and Welfare, *OR*, odds ratio, PDRC, Panel Data Research Center, SES, socioeconomic status, TIPI-J, Japanese version of the Ten Item Personality Inventory, Personality traits, Health behavior, Working women

## Abstract

Considering personality traits is effective for cultivating health promotion habits. Therefore, individualized interventions that account for personality traits would be more beneficial for modifying health behaviors. The present study describes the relationship between personality traits and health behaviors among working women compared with non-working women in Japan. Secondary analysis was conducted using nationally representative data from the Japanese Household Panel Survey (JHPS/KHPS). In the 2019 wave, data were gathered from 1,141 women (939 who were working and 202 who were not) between the ages of 28 and 65. Associations among health behaviors, personality traits, and employment status were confirmed by phi coefficient and coefficient ratio. Logistic regression analysis was conducted to examine the association between health behaviors and personality traits. As for working women, daily fruit consumption was associated with extraversion (odds ratio [*OR*], 1.197; *p* = 0.003) and conscientiousness (*OR*, 1.238; *p* = 0.032). Conscientiousness has been found to significantly contribute to low-risk alcohol consumption (OR, 1.213; p = 0.035). Desirable physical activity habit was associated with extraversion (*OR,* 1.312; *p* = 0.000). In contrast, among non-working women, desirable physical activity habits was associated with extraversion (OR, 1.573; *p* = 0.007) and neuroticism (OR, 0.390; *p* = 0.001). Further research is needed to clarify the mechanisms underlying differences between working women and non-working women in the association between health behaviors and personality.

## 1 Introduction

In recent years, women have increasingly participated in the labor force around the world. In Japan, the aging population and the shrinking working population have promoted the participation of women in the labor market, and the Cabinet Office has proposed a priority policy to support social participation (Health and Global Policy Institute, 2018).

From the perspective of biological and social differences, women need to pay particular attention to their health. In fact, health promotion strategies are still inadequate in terms of enabling women to be active in the workplace (Health and Global Policy Institute, 2018). For working women to be healthy and active both physically and mentally, they must manage their health behaviors. However, many working women in Japan are likely to exhibit unfavorable health behaviors and lifestyle habits (Health and Global Policy Institute, 2018, Ministry of Health, Labour and Welfare, 2021a). In addition to interventions for environmental and psychosocial factors, which have been explored to improve health behaviors (Agosti et al., 2019), other findings are needed for more optimal individual interventions to modify health behaviors (Roberts et al., 2007).

In recent years, studies have also focused on personality as an important factor related to health behaviors such as dietary intake, smoking habits, alcohol consumption, and physical exercise (Rhodes and Smith, 2006, Takahashi et al., 2013). Previous studies have suggested that individualized interventions that account for personality traits are more beneficial for modifying health behaviors (Magidson et al., 2014, Wilson and Dishman, 2015). Cultivating health promotion habits that consider individuals’ personality traits would be effective. However, there is little research on the association between personality and health promotion behaviors in Japan, especially among working women.

Therefore, this study aimed to describe the relationship between personality traits and health behaviors among working women compared with non-working women in Japan.

## 2 Methods

### 2.1 Data

Secondary analysis was conducted using nationally representative data from the Japanese Household Panel Survey (JHPS/KHPS) by the Panel Data Research Center (PDRC) at Keio University (Keio University, n.d). KHPS has been conducted annually since 2004 and surveys 4,005 men and women born between 1935 and 1984; JHPS has been conducted annually since 2009 and surveys 4,000 men and women born after 1927. JHPS/KHPS use a stratified two-stage sampling method.

### 2.2 Participants

The sample of this study was based on data collected from women between the ages of 28 and 65 from the 2019 wave of JHPS/KHPS. Eligibility criteria for the sample analyzed in this study were (1) age up to 65 years (age 65 years is the upper limit of Japan's working-age population) and (2) no missing responses to survey items used in the analysis.

### 2.3 Variables

#### 2.3.1 Personality

Personality was assessed using the Ten Item Personality Inventory Japanese version (TIPI-J) (Gosling et al., 2003, Oshio et al., 2012). TIPI-J measures five domains originating from the Big Five theory (Gosling et al., 2003), namely, Extraversion (i.e., proactive, sociable, and self-disciplined), Agreeableness (i.e., cooperative, sympathetic, and trustworthy), Conscientiousness (i.e., disciplined, responsible, self-controlled, and goal-oriented), Neuroticism (i.e., anxious, impulsive, and insecure), and Openness (i.e., intellectually curious, imaginative, and creative). Each item is rated on a seven-point Likert scale (from 1*, “*strongly agree,” to 7, *“*strongly disagree”). Higher scores reflect a higher level of the trait.

#### 2.3.2 Health behaviors

Health behavior variables were alcohol consumption (1 = < 23 g per day or 0 ≥ 23 g per day), smoking status (1 = not smoking at present or 0 = smoking), vegetable and fruit consumption (1 = every day or 0 = not every day), and physical activity (1 = more than 2 days or 0 = 0 to 1 day). These health behaviors were found to be beneficial in maintaining and improving health by the World Health Organization (WHO), Ministry of Health, Labour and Welfare (MHLW) in Japan, and other previous studies (e.g., Breslow and Enstrom, 1980, Ministry of Health, Labour and Welfare, 2021a). Each variable was coded based on standards recommended in previous studies and reports by the WHO and MHLW (Breslow and Enstrom, 1980, Ministry of Health, Labour and Welfare, 2021a).

#### 2.3.3 Sociodemographic variables

Sociodemographic variables were age, sex (male or female), marital status (married, not married, or separated/bereaved), academic background (junior or senior high school graduate; junior college, university, or graduate school degree; or other), employment status (regularly employed, non-regularly employed, self-employed, and non-working), and socioeconomic status (SES; expressed in increments of 10,000 yen). As for SES, equivalent disposable income was calculated and participants were divided into “higher” and “lower” groups based on the poverty line in Japan (equivalent disposable income of 122,000 yen per year).

### 2.4 Analysis

The data were analyzed using SPSS 25.0 for Mac. Descriptive statistics for all variables were calculated. Next, phi coefficient confirmed the association between health behaviors and employment status. Correlation ratios (η square) were also calculated to confirm the association between employment status and personality traits. Finally, logistic regression was conducted to clarify the association between health behaviors and personality traits for each participant group (working women and non-working women). Statistical significance was set at *p* < 0.05 (two-tailed).

### 2.5 Ethical considerations

The JHPS/KHPS data used in this study were provided by PDRC (#2527). The data for secondary analysis were completely anonymized and provided by PDRC. Ethical approval was not required because this study used only secondary analysis (Ministry of Health, Labour and Welfare, 2021b). Consent was obtained from all participants in the JHPS/KHPS regarding the purpose of the research, potential use of their data, data anonymity, strict protection of individual data, and secondary use of data by PDRC.

## 3 Results

The study sample included 939 working women and 202 non-working women. In working women, the mean age was 49.87 ± 8.75 years, 60.8% were non-regularly employed, and 28.9% were regularly employed. In non-working women, the mean age was 53.34 ± 8.77 years (Table 1). Alcohol consumption habits were related to employment status (phi coefficient = -0.114, *p* = 0.000). No significant associations were found between personality trait and employment status (see Appendix 1).

**Table 1:** Sample characteristics.

		Working (*n* = 939)		Non-working (*n* = 202)	
		*N*	%	*N*	%
Marital status					
Not married		167	17.8	14	6.9
Separated/bereaved		22	2.3	0	0.0
Married		750	79.9	188	93.1

Academic background					
Junior or senior high school graduate		383	40.8	100	49.5
Junior college, university, or graduate degree		468	49.8	88	43.6
Other		88	9.4	14	6.9

SES*					
Lower		378	40.3	108	53.5
Higher		561	59.7	94	46.5

Employment status					
Regular		271	28.9	–	–
Nonregular		571	60.8	–	–
Self-employed		97	10.3	–	–
Non-working		–	–	202	100

		Working (*n* = 939)		Non-working (*n* = 202)	
		Mean	*SD*	Mean	*SD*
Extraversion		4.30	1.27	3.96	1.28
Agreeableness		4.98	0.90	5.04	0.87
Conscientiousness		4.10	0.77	4.35	0.87
Neuroticism		3.94	0.80	3.98	0.82
Openness		4.01	0.79	4.17	0.84
Age		49.87	8.75	53.34	8.77

*SES: socioeconomic status

In working women, daily fruit consumption was associated with extraversion (odds ratio [*OR*], 1.197; 95% confidence interval [CI], 1.065–1.345; *p* = 0.003) and conscientiousness (*OR*, 1.238; 95% CI, 1.019–1.504; *p* = 0.032). Low-risk alcohol consumption was associated with conscientiousness (*OR*, 1.213; 95% CI, 1.014–1.453; *p* = 0.035). Desirable physical activity habits was associated with extraversion (*OR*, 1.312; 95% CI, 1.132–1.521; *p* = 0.000) (Table 2).

**Table 2:** Logistic regression analysis for health behaviors: working women.

	Smoking habit (ref. smoker)					Alcohol consumption (ref. ≥ 23gram)					Physical activity (ref. 0 to 1 day)				
	*OR*	*SE*	95% CI		*P*	*OR*	*SE*	95% CI		*P*	*OR*	*SE*	95% CI		*p*
			Lower	Upper				Lower	Upper				Lower	Upper	
Extraversion	0.851	0.093	0.710	1.021	0.083	0.904	0.055	0.812	1.006	0.065	1.312	0.075	1.132	1.521	0.000
Agreeableness	1.102	0.123	0.867	1.401	0.429	0.922	0.076	0.794	1.070	0.285	1.216	0.106	0.989	1.497	0.064
Conscientiousness	1.234	0.151	0.919	1.659	0.162	1.213	0.092	1.014	1.453	0.035	1.032	0.125	0.807	1.319	0.803
Neuroticism	1.011	0.152	0.751	1.361	0.943	0.877	0.090	0.734	1.046	0.144	0.795	0.123	0.624	1.012	0.063
Openness	0.957	0.146	0.718	1.274	0.761	1.084	0.089	0.911	1.290	0.363	0.834	0.121	0.658	1.056	0.131
Academic background (ref. junior or senior high school graduate)															
Junior college, university, or graduate degree	4.969	0.271	2.920	8.456	0.000	0.955	0.143	0.721	1.265	0.749	1.526	0.198	1.036	2.248	0.032
Other	2.660	0.418	1.172	6.039	0.019	1.189	0.242	0.741	1.909	0.473	1.539	0.319	0.823	2.878	0.177
SES* (ref. lower)	0.953	0.239	0.597	1.521	0.839	0.878	0.140	0.667	1.155	0.352	1.067	0.191	0.733	1.552	0.736
Marital status (ref. single)															
Separated/bereaved	2.004	0.680	0.528	7.600	0.307	0.811	0.476	0.319	2.063	0.661	1.663	0.576	0.537	5.144	0.378
Married	4.427	0.253	2.695	7.272	0.000	1.186	0.180	0.834	1.687	0.342	0.975	0.248	0.599	1.587	0.919
Employment status (ref. regular)															
Nonregular	1.203	0.261	0.721	2.007	0.480	0.955	0.157	0.702	1.298	0.768	1.637	0.230	1.042	2.570	0.032
Self-employed	1.296	0.430	0.558	3.011	0.546	0.805	0.246	0.497	1.305	0.378	2.612	0.309	1.427	4.784	0.002
Age	1.005	0.014	0.978	1.032	0.731	0.994	0.008	0.979	1.010	0.477	1.023	0.011	1.001	1.045	0.038
Nagelkerke *R*^2^	0.182					0.024					0.073				

	Vegetable consumption (ref. not every day)					Fruit consumption (ref. not every day)				
	*OR*	*SE*	95% CI		*p*	*OR*	*SE*	95% CI		*p*
			Lower	Upper				Lower	Upper	
Extraversion	0.955	0.074	0.826	1.103	0.531	1.197	0.060	1.065	1.345	0.003
Agreeableness	1.025	0.101	0.841	1.250	0.803	0.860	0.082	0.732	1.010	0.066
Conscientiousness	1.085	0.123	0.854	1.380	0.505	1.238	0.099	1.019	1.504	0.032
Neuroticism	1.012	0.121	0.798	1.282	0.924	0.832	0.097	0.688	1.006	0.058
Openness	1.036	0.119	0.821	1.308	0.767	0.974	0.095	0.809	1.173	0.782
Academic background (ref. junior or senior high school graduate)										
Junior college, university, or graduate degree	1.880	0.193	1.287	2.747	0.001	1.872	0.156	1.379	2.542	0.000
Other	1.951	0.353	0.977	3.895	0.058	1.933	0.256	1.171	3.190	0.010
SES* (ref. lower)	1.365	0.188	0.945	1.972	0.097	0.975	0.150	0.726	1.308	0.864
Marital status (ref. not married)										
Separated/bereaved	0.811	0.523	0.291	2.258	0.688	0.988	0.518	0.358	2.725	0.981
Married	1.839	0.219	1.197	2.826	0.005	1.326	0.196	0.903	1.947	0.150
Employment status (ref. regular)										
Nonregular	1.072	0.214	0.705	1.630	0.746	0.771	0.167	0.556	1.069	0.119
Self-employed	0.927	0.326	0.489	1.756	0.815	1.177	0.252	0.719	1.928	0.516
Age	0.996	0.011	0.976	1.018	0.742	1.051	0.009	1.033	1.069	0.000
Nagelkerke *R*^2^	0.063					0.096				

*SES: socioeconomic status; OR: odds ratio; SE: 95% CI: 95% confidence interval.

On the other hand, among non-working women, desirable physical activity habits was associated with extraversion (OR, 1.573; 95% CI, 1.134–2.180; *p* = 0.007) and neuroticism (*OR*, 0.390; 95% CI, 0.220–0.691; *p* = 0.001) (see Appendix 2).

## 4 Discussion

The present study demonstrated that among Japanese working women, higher extraversion was associated with desirable physical activity habit and daily fruit consumption and higher conscientiousness was associated with low-risk alcohol consumption habits and daily fruit consumption. However, non-working women exhibited higher extraversion and lower neuroticism in association with desirable physical activity habits, in line with previous research (Kekäläinen et al., 2020). This difference might depend on personality trait, employment status, or other individual factors. However, despite discussion about the importance of personality traits in relation to attempted health behavior changes, there remains a paucity of studies about this topic. Thus, while further investigations are needed, our findings will contribute to the current debate on strategies for health behavior change.

Previous research using larger samples have reported that personality traits play a significant role in adaptive health behaviors such as nonsmoking, low-risk alcohol consumption, healthy eating habits, and physical activity (Bogg and Roberts, 2004, Keller and Siegrist, 2015, Rhodes and Smith, 2006). People with high extraversion and conscientiousness are more likely to engage in healthy dietary behaviors, and higher conscientiousness is also significantly associated with lower alcohol use (Hakulinen et al., 2015, Keller and Siegrist, 2015, Lunn et al., 2014). People with high conscientiousness have a high level of awareness, responsibility, and compliance regarding their health promotion behaviors. Although this study population was limited to working women, the findings of this study support those of previous studies.

Consuming greater than 200 g of fruit per day on average is recommended for improved health, but workers may have lower fruit consumption (Ministry of Health, Labour and Welfare, 2021a). Japanese working women operate in a stressful work environment in terms of both quality and quantity, and they have insufficient time to reflect on their own health behaviors. Rodrigues et al. (2021) indicated that stressful work conditions can decrease awareness of fruit intake. In such a situation, those who have a desirable habit of consuming fruits are highly aware of their own health care.

In addition, Japanese workers tend to drink alcohol frequently while socializing with their business partners. Even under such circumstances, women with higher conscientiousness, assumed to be aware of their own health, avoid risky drinking behaviors.

Since extraversion refers to individual tendencies to be proactive, sociable, and self-disciplined (Gosling et al., 2003, Oshio et al., 2012), people with high extraversion are more likely to adhere to desirable fruit eating habits and maintain exercise habits (Bogg and Roberts, 2004). Moreover, extraversion has an energetic aspect and active tendency, which explains the significant positive association between extraversion and participation in physical activity (Rhodes and Smith, 2006). This study indicated that high extraversion and self-discipline can also be positively reflected in Japanese women’s attitudes toward positive health behaviors.

This study has a number of limitations. First, although other countries have reported health behavior characteristics by gender, very few have reported characteristics by employment status, thus limiting interpretation of the characteristics of this sample. Furthermore, no literature has reported personality characteristics by employment status. Therefore, once new findings have accumulated in the future, the sample characteristics in this study can be interpreted appropriately.

Second, the 2019 KHPS/JHPS survey provided the baseline of personality traits; therefore, this is a preliminary study to investigate the relationship between personality and health behavior based on cross-sectional data. Further study will be conducted more rigorously to clarify causal relationships or mechanisms between personality traits and health behaviors using longitudinal data on personality traits.

Third, the data on health behaviors relied on self-reported information, so responses might reflect social desirability bias. Observational tools for measuring health behaviors should be used. Fourth, our exploratory study should have considered other psychosocial factors or personality variables. Finally, the age of the analyzed sample range is limited owing to use of secondary data and a certain percentage of participants dropping out or being excluded because of missing responses. Therefore, further research with a stratified sampling of age and employment status would be recommended. Moreover, generalization of our results to other countries with different socioeconomic and cultural backgrounds should be interpreted with caution.

## 5 Conclusion

This study demonstrated that higher extraversion was significantly associated with desirable physical activity habits and daily fruit consumption, and higher conscientiousness was significantly associated with low-risk alcohol consumption habits and daily fruit consumption among working women in Japan.

### CRediT authorship contribution statement

**Miho Satoh:** Conceptualization, Methodology, Data curation, Writing – original draft, Investigation, Project administration. **Naoko Sato:** Conceptualization, Methodology, Writing – review & editing, Supervision. **Akiko Fujimura:** Conceptualization, Methodology, Writing – review & editing.

## Declaration of Competing Interest

The authors declare that they have no known competing financial interests or personal relationships that could have appeared to influence the work reported in this paper.
