# Supplementary material for: Relationship between personality traits and health behavior among working women in Japan
Source: Prev Med Rep. 2021 Dec 27;25:101691. doi: 10.1016/j.pmedr.2021.101691 (PMC8800055; doi:10.1016/j.pmedr.2021.101691)
Supplement: Supplementary data 1 [file mmc1.docx]

Table A.1. Association Among Health Behaviors, Personality Traits, and Employment Status

|  |  | Working  (*n* = 939) | |  | Non-working  (*n* = 202) | |  | χ2 | φ | *p* |
| --- | --- | --- | --- | --- | --- | --- | --- | --- | --- | --- |
|  |  | *N* | % |  | *N* | % |  |  |  |  |
| Smoking habit | |  |  |  |  |  |  | 1.796 | -0.040 | 0.180 |
|  | Non-smoking | 840 | 89.5 |  | 187 | 92.6 |  |  |  |  |
|  | Smoker | 99 | 10.5 |  | 15 | 7.4 |  |  |  |  |
|  |  |  | |  |  | |  |  |  |  |
| Alcohol consumption | |  |  |  |  |  |  | 14.956 | -0.114 | 0.000 |
|  | ＜23gram | 431 | 45.9 |  | 123 | 60.9 |  |  |  |  |
|  | ≥ 23gram | 508 | 54.1 |  | 79 | 39.1 |  |  |  |  |
|  |  |  | |  |  | |  |  |  |  |
| Physical activity | |  |  |  |  |  |  | 0.461 | -0.020 | 0.497 |
|  | More than 2 days | 158 | 16.8 |  | 38 | 18.8 |  |  |  |  |
|  | 0 to 1 day | 781 | 83.2 |  | 164 | 81.2 |  |  |  |  |
|  |  |  | |  |  | |  |  |  |  |
| Vegetable consumption | |  |  |  |  |  |  | 0.025 | -0.005 | 0.874 |
|  | Every day | 786 | 83.7 |  | 170 | 84.2 |  |  |  |  |
|  | Not every day | 153 | 16.3 |  | 32 | 15.8 |  |  |  |  |
|  |  |  |  |  |  |  |  |  |  |  |
| Fruit consumption | |  |  |  |  |  |  | 3.670 | -0.057 | 0.055 |
|  | Every day | 337 | 35.9 |  | 87 | 43.1 |  |  |  |  |
|  | Not every day | 602 | 64.1 |  | 115 | 56.9 |  |  |  |  |
|  | | Working  (*n* = 939) | |  | Non-working  (*n* = 202) | |  | η |  |  |
|  | | Mean | *SD* |  | Mean | *SD* |  |  |  |  |
| Extraversion | | 4.30 | 1.27 |  | 3.96 | 1.28 |  | 0.010 |  |  |
| Agreeableness | | 4.98 | 0.90 |  | 5.04 | 0.87 |  | 0.001 |  |  |
| Conscientiousness | | 4.10 | 0.77 |  | 4.35 | 0.87 |  | 0.015 |  |  |
| Neuroticism | | 3.94 | 0.80 |  | 3.98 | 0.82 |  | 0.000 |  |  |
| Openness | | 4.01 | 0.79 |  | 4.17 | 0.84 |  | 0.006 |  |  |
| Age | | 49.87 | 8.75 |  | 53.34 | 8.77 |  | 0.022 |  |  |

Table A.2. Logistic Regression Analysis for Health Behaviors: Non-Working Women

|  |  | Smoking habit (ref. smoker) | | | | | |  | Alcohol consumption (ref. ≥ 23gram) | | | | |  | Physical activity (ref. 0 to 1 day) | | | | | | | |
| --- | --- | --- | --- | --- | --- | --- | --- | --- | --- | --- | --- | --- | --- | --- | --- | --- | --- | --- | --- | --- | --- | --- |
|  |  | *OR* | *SE* | 95% CI | | | *p* |  | *OR* | *SE* | 95% CI | | *p* |  | *OR* | *SE* | | 95% CI | | | *p* | |
|  |  |  |  | Lower | | Upper |  |  |  |  | Lower | Upper |  |  |  | |  | | Lower | Upper | |  |
| Extraversion | | 0.771 | 0.247 | 0.475 | | 1.251 | 0.292 |  | 0.973 | 0.123 | 0.764 | 1.238 | 0.822 |  | 1.573 | 0.167 | | 1.134 | | 2.180 | 0.007 | |
| Agreeableness | | 0.889 | 0.350 | 0.448 | | 1.764 | 0.736 |  | 1.096 | 0.186 | 0.761 | 1.580 | 0.622 |  | 0.739 | 0.259 | | 0.445 | | 1.228 | 0.243 | |
| Conscientiousness | | 1.708 | 0.385 | 0.803 | | 3.634 | 0.165 |  | 1.006 | 0.188 | 0.696 | 1.455 | 0.973 |  | 1.286 | 0.249 | | 0.789 | | 2.097 | 0.312 | |
| Neuroticism | | 0.780 | 0.368 | 0.379 | | 1.603 | 0.499 |  | 1.478 | 0.208 | 0.984 | 2.220 | 0.060 |  | 0.390 | 0.292 | | 0.220 | | 0.691 | 0.001 | |
| Openness | | 0.753 | 0.339 | 0.387 | | 1.464 | 0.403 |  | 1.414 | 0.198 | 0.960 | 2.084 | 0.080 |  | 0.607 | 0.276 | | 0.353 | | 1.041 | 0.070 | |
| Academic background (ref. junior or senior high school graduate) | | | | | | | |  |  |  |  |  |  |  |  |  | |  | |  |  | |
|  | Junior college, university, or graduate degree | 5.638 | 0.802 | 1.170 | | 27.157 | 0.031 |  | 0.599 | 0.318 | 0.321 | 1.118 | 0.108 |  | 2.662 | 0.426 | | 1.154 | | 6.140 | 0.022 | |
|  | Other | 2.011 | 1.163 | 0.206 | | 19.646 | 0.548 |  | 0.342 | 0.605 | 0.105 | 1.120 | 0.076 |  | 0.614 | 1.182 | | 0.060 | | 6.233 | 0.680 | |
| SES^*^ (ref. lower） | | 1.516 | 0.598 | 0.470 | | 4.891 | 0.486 |  | 0.828 | 0.305 | 0.456 | 1.504 | 0.536 |  | 1.478 | 0.415 | | 0.655 | | 3.336 | 0.346 | |
| Marital status (ref. single） | |  |  |  | |  |  |  |  |  |  |  |  |  |  |  | |  | |  |  | |
| Married | | 1.729 | 0.877 | 0.310 | | 9.653 | 0.532 |  | 0.979 | 0.607 | 0.298 | 3.219 | 0.972 |  | 1.357 | 0.883 | | 0.240 | | 7.667 | 0.729 | |
| Age | | 0.992 | 0.036 | 0.925 | | 1.065 | 0.833 |  | 0.993 | 0.018 | 0.958 | 1.029 | 0.685 |  | 1.084 | 0.027 | | 1.027 | | 1.144 | 0.003 | |
| Nagelkerke *R*^2^ | | 0.161 |  | |  |  |  |  | 0.091 |  |  |  |  |  | 0.250 |  | |  | |  |  | |

*SES: socioeconomic status; OR: odds ratio; S*E*: 95% CI: 95% confidence interval

Table A.2. Logistic Regression Analysis for Health Behaviors: Non-Working Women (cont.)

|  |  | Vegetable consumption  (ref. not every day) | | | | |  | Fruit consumption (ref. not every day) | | | | |
| --- | --- | --- | --- | --- | --- | --- | --- | --- | --- | --- | --- | --- |
|  |  | *OR* | *SE* | 95% CI | | *p* |  | *OR* | *SE* | 95% CI | | *p* |
|  |  |  |  | Lower | Upper |  |  |  |  | Lower | Upper |  |
| Extraversion | | 1.203 | 0.178 | 0.849 | 1.704 | 0.299 |  | 1.191 | 0.132 | 0.919 | 1.543 | 0.187 |
| Agreeableness | | 1.460 | 0.254 | 0.887 | 2.404 | 0.137 |  | 0.790 | 0.198 | 0.536 | 1.164 | 0.233 |
| Conscientiousness | | 0.637 | 0.252 | 0.389 | 1.044 | 0.073 |  | 1.044 | 0.202 | 0.703 | 1.549 | 0.832 |
| Neuroticism | | 0.839 | 0.264 | 0.499 | 1.408 | 0.505 |  | 0.694 | 0.216 | 0.455 | 1.059 | 0.090 |
| Openness | | 1.411 | 0.259 | 0.848 | 2.346 | 0.185 |  | 0.902 | 0.206 | 0.603 | 1.350 | 0.617 |
| Academic background (ref. junior or senior high school graduate) | | | | | | |  |  |  |  |  |  |
|  | Junior college, university, or graduate degree | 2.622 | 0.450 | 1.085 | 6.336 | 0.032 |  | 1.972 | 0.345 | 1.003 | 3.877 | 0.049 |
|  | Other | 522933694.595 | 10454.484 | 0.000 | . | 0.998 |  | 1.760 | 0.650 | 0.492 | 6.298 | 0.385 |
| SES^*^ (ref. lower） | | 0.880 | 0.418 | 0.387 | 1.997 | 0.759 |  | 0.927 | 0.325 | 0.490 | 1.752 | 0.814 |
| Marital status (ref. not married） | |  |  |  |  |  |  |  |  |  |  |  |
| Married | | 2.283 | 0.677 | 0.606 | 8.603 | 0.223 |  | 0.521 | 0.702 | 0.132 | 2.062 | 0.353 |
| Age | | 1.017 | 0.024 | 0.969 | 1.067 | 0.492 |  | 1.129 | 0.023 | 1.080 | 1.180 | 0.000 |
| Nagelkerke *R*^2^ | | 0.164 |  |  |  |  |  | 0.258 |  |  |  |  |

*SES: socioeconomic status; OR: odds ratio; SE: 95% CI: 95% confidence interval
